# Family cash transfers in childhood and birthing persons and birth outcomes later in life

**DOI:** 10.1016/j.ssmph.2024.101623

**Published:** 2024-02-15

**Authors:** Brenda Bustos, Marcela Lopez, Kenneth A. Dodge, Jennifer E. Lansford, William E. Copeland, Candice L. Odgers, Tim A. Bruckner

**Affiliations:** a Program in Public Health, University of California, Irvine, 856 Health Sciences Quad Irvine, CA, 92697, USA; b Department of Epidemiology and Biostatistics, University of California, Irvine, 856 Health Sciences Quad Irvine, CA, 92697, USA; c Sanford School of Public Policy, Duke University, 201 Science Drive, Durham, NC, 27708, USA; d Department of Psychiatry, University of Vermont, 1 South Prospect, Burlington, VT, 05405, USA; e School of Social Ecology, University of California, Irvine, 4326 Social & Behavioral Sciences Gateway, Irvine, CA, 92697, USA; f Center for Population, Inequality, and Policy, University of California, Irvine, School of Social Sciences, Irvine, CA, 92697, USA

**Keywords:** Cash transfers, Intergenerational transmission, Birth outcomes

## Abstract

Much literature in the US documents an intergenerational transmission of birthing person and perinatal morbidity in socioeconomically disadvantaged groups. A separate line of work indicates that family cash transfers may improve life chances of low-income families well into adulthood. By exploiting a quasi-random natural experiment of a large family cash transfer among a southeastern American Indian (AI) tribe in rural North Carolina, we examine whether a “perturbation” in socioeconomic status during childhood improves birthing person/perinatal outcomes when they become parents themselves. We acquired birth records on 6805 AI and non-AI infants born from 1995 to 2018. Regression methods to examine effect modification tested whether the birthing person's American Indian (AI) status and exposure to the family cash transfer during their childhood years corresponds with improvements in birthing person and perinatal outcomes. Findings show an increase in age at childbearing (coef: 0.15 years, 95% confidence interval [CI]: 0.05, 0.25) and a decrease in pre-pregnancy body mass index (BMI; coef: −0.42, 95% CI: −0.76, −0.09) with increased duration of cash transfer exposure during childhood. The odds of large-for-gestational age at delivery, as well as mean infant birthweight, is also reduced among AI births whose birthing person had relatively longer duration of exposure to the cash transfer. We, however, observe no relation with other birthing person/perinatal outcomes (e.g., tobacco use during pregnancy, preterm birth). In this rural AI population, cash transfers in one generation correspond with improved birthing person and infant health in the next generation.

## Highlights

• We examined a large family cash transfer among an American Indian (AI) tribe in rural North Carolina.
• AI birthing persons show an increase in age at childbearing with increased cash transfer exposure as a child.
• AI birthing persons show a decrease in pre-pregnancy body mass index with increased cash transfer exposure as a child.
• For several birth outcomes, large cash transfers during childhood may improve health into the subsequent generation.

## Introduction

In the US, parents' socioeconomic status (SES) strongly contributes to children's life chances (Chetty et al., 2017; Hällsten & Thaning, 2022; Odgers, 2015). These life chances refer to factors such as educational attainment, health, and financial well-being into adulthood (Akee et al., 2010; Copeland et al., 2022; Costello et al., 2010). Much theory and empirical work, moreover, contends that the social and economic environment that individuals are exposed to across childhood and adolescence affects the future birthing person's health outcomes, and their perinatal outcomes, later in life (Lu et al., 2010; Lu & Halfon, 2003; Pearl et al., 2018).

Given increasing recognition of the strong intergenerational transmission of disadvantage and the lack of upward socioeconomic mobility among low-income families (Chetty, Hendren, Kline, Saez, & Turner, 2014), scholars and policymakers have proposed direct cash transfers (e.g., a child tax credit (Aizer et al., 2022)) to boost the financial resources of low-income families with children. Cash transfer programs in the US take quite diverse forms but tend to receive funding by the federal government and target low-income families with children. Such proposals assume that cash transfers during childhood will, through myriad pathways, improve health and human capital well into adulthood (Sun et al., 2021). These pathways include improvements in material and environmental conditions, psychological gains, changes in health behaviors, parental availability, educational investments, and increased access to medical care.

In the late 1990s, the Eastern Band of Cherokee Indians (EBCI) in rural North Carolina underwent a natural experiment by way of the introduction of a casino on their lands. Under the terms of the agreement, the casino would allocate a percentage of profits to all enrolled EBCI every year. Since 1996, gaming proved profitable and per capita payments from the casino to EBCI averaged approximately $5000 per year. Poverty fell precipitously among the EBCI after the introduction of the per capita payment (i.e., from almost 60% to <25% in five years) (Costello et al., 2003). Prior studies also found improved educational attainment, mental health, and financial well-being into adulthood among EBCI whose families received cash transfers during their childhood (relative to later in life) (Akee et al., 2010; Copeland et al., 2022; Costello et al., 2010). Importantly, findings show a larger effect size when children living at home were younger when their EBCI families received the transfers (Copeland et al., 2022).

The quasi-random timing of the large family cash transfer during childhood among the EBCI allows us to examine whether such “perturbations” in SES affect subsequent outcomes of the birthing person (e.g., age at childbearing, body mass index, educational attainment) and their infant (e.g., birthweight, preterm delivery, weight-for-gestational age). Improvements in childhood material, environmental, or psychosocial conditions, for instance, could alter health behaviors, educational attainment, fertility timing, and access to medical care—all of which could affect outcomes later in life (Copeland et al., 2022, Heckman, 2000, Mallar, 1977; Maynard, 1977; Maynard & Murnane, 1979). In this study we test whether these assumed improvements, consistent with the theory of human capital formation (Heckman, 2000), would extend even further to the birthing person's outcomes and perinatal outcomes of the next generation. We view this study as an important contribution to the field because it informs whether childhood human capital interventions have the potential to reduce or break intergenerational transmission of disadvantage in birthing person's and perinatal outcomes.

## Methods

### Study population

We examined American Indians (AI) in Jackson, Swain, and Graham counties in North Carolina as recipients of a family cash transfer available to the Eastern Band of Cherokee Indians (EBCI). Previous studies have used the census indicator of AI as a proxy for EBCI in this region (Kaijser et al., 2009). Although birth records do not capture an AI birthing person's tribal identification, we can presume their Cherokee status because no other Tribes have federally or state recognized claimed land in this area.

AI residents in this area received the family cash transfer beginning in 1996. Non-AI residents, by contrast, did not receive family cash transfers. We, as described below, use non-AI birthing persons in Jackson, Swain, and Graham counties as another comparison group and therefore include non-AI births in the study population.

### Variables and data

We acquired birth records from the Birth File of the North Carolina Office of Vital Records. The Birth File extracts information from birth certificates including a small set of health and sociodemographic characteristics of the birthing parent and health characteristics of the infant. We restricted our sample to live-born infants in Jackson, Swain, and Graham counties to AI and non-AI birthing persons aged 7–17 years in 1995. Birthing persons in this age group in 1995 may be exposed before age 18 to the cash transfer from 0 to 10 years. In addition, this sample has the opportunity to age to at least 30 years by 2018 and yield a live birth—the last year of our birth data—which permits analysis of differences in birth outcomes as a function of our exposure, for a substantial portion of the population's reproductive history. This restriction yielded 3852 birthing persons who birthed a total of 6805 children from 1995 to 2018 (Table 1).

**Table 1:** Birthing person and birth characteristics of American Indian (AI) and non-American Indian (Non-AI) infants in Jackson, Swain, and Graham counties born from 1997 to 2018.

	AI		Non-AI	
	N	%^a^	N	%^a^
Birthing Person's Age (years)				
17 or younger	159	9.7	224	4.3
18 to 24	873	53.0	2223	43.1
25 to 29	427	25.9	1612	31.3
30 or older	188	14.6	1099	21.3
**Birthing Person's Education**				
Less than high school	503	30.5	1124	21.8
High school graduate	628	38.1	1389	26.9
>High School	423	25.7	2301	44.6
**Married**				
Yes	555	33.7	3384	65.6
No	1090	66.2	1774	34.4
**Birthing Person's BMI Prior to Pregnancy**^b^				
<24.99	91	20.3	635	34.1
25–29.99	129	28.8	479	25.7
30–34.99	86	19.2	272	14.6
35–39.99	57	12.7	148	7.9
40–50	46	10.3	82	4.4
**Tobacco Use During Pregnancy**				
Yes	403	24.5	1115	21.6
No	1153	70.0	3702	71.8
**Infant Sex**				
Male	838	50.9	2689	52.1
Female	809	49.1	2469	47.9
**Birthweight (grams)**				
500–1499	19	1.2	61	1.2
1500–1999	9	0.6	67	1.3
2000–2499	62	3.8	241	4.7
2500–2999	277	16.8	869	16.9
3000–3999	1053	63.9	3458	67.0
4000–5999	225	13.7	455	8.8
**Preterm Birth (**<**37 weeks)**				
Yes	140	8.7	411	8.2
No	1471	91.3	4624	91.8
	**Mean**	**SD**	**Mean**	**SD**
Birthing Person's Age (years)	23.3	4.7	25.2	5.0
Birthing Person's BMI Prior to Pregnancy^b^	29.9	7.6	27.2	6.7
Birthweight (grams)	3387.8	607.1	3299.6	581.6

a Column percents may not sum to 100 due to missing values for that variable.

b Data collection for these variables began in 2011.

In line with previous literature, we identified our exposure among AI persons as the childhood years remaining before reaching age 18 (Copeland et al., 2022; Costello et al., 2003). Prior literature finds a positive relation between the number of years exposed to the family cash transfer during childhood and later-life health among AI persons (Copeland et al., 2022). Hence, we presume that the birthing person's age when the family cash transfer began may influence subsequent birthing person/perinatal health and the timing of childbearing. As a result, our exposure is measured as the interaction between a birthing person's AI status and the number of childhood years remaining after 1996 (AI*duration).

Improvements in childhood SES by way of the family cash transfer could alter health behaviors, educational attainment, fertility timing, and access to medical care—all of which could affect birthing person/perinatal outcomes. We therefore retrieved several variables related to the birthing person and the birth outcome which could plausibly respond to different levels of cash transfer exposure in childhood. These variables may reflect favorable socioeconomic birthing person outcomes (e.g., later age at childbearing, greater educational attainment) as well as improved perinatal outcomes (e.g., reduced risk of high/low birthweight and preterm birth, normative fetal growth). Relevant outcome variables in the birth file include age at childbirth (continuous, in years), highest educational attainment prior to pregnancy (1 = less than high school, 2 = high school graduate, and 3 = more than high school), pre-pregnancy body mass index (BMI; kg/m^2^), tobacco use during pregnancy (yes/no), infant's birthweight (in grams), preterm birth (=1 if delivery <37 weeks; = 0 otherwise), small-for-gestational age (SGA; = 1 if less than 10^th^ percentile of weight-for-gestational week using INTERGROWTH-21 reference tables; = 0 otherwise), and large-for-gestational age (LGA; restricted analysis only to term births, coded as “1” if greater than 90^th^ percentile of weight-for-gestational week [Villar et al., 2014]; and coded “0” otherwise).

AI birthing persons in the US, who have greater mean pre-pregnancy BMI than do other race/ethnicities, also show the greatest risk of delivering a heavier than average (i.e., macrosomic) birth (Anderson et al., 2016). Macrosomic births (typically defined as >4000 g) appear more likely than normal weight births to exhibit obesity and diabetes (Leddy et al., 2008; Lindberg et al., 2012; Whincup et al., 2008) later in life. We therefore examined the possibility that, among births not considered low weight (i.e., <2500 gms) the family cash transfer might vary inversely with birth weight, thereby reducing the risk of macrosomia.

### Analysis

AI residents in this area received the family cash transfer beginning in 1996. This large cash transfer averages about $5000 per year per adult 21 years and older (or 18 years and older contingent on having graduated high school). We employ a regression strategy to isolate potential benefits of the family cash transfer on future birthing person outcomes/perinatal outcomes of AI children who were young in 1996—the first year of the family cash transfer program. This approach, which focuses on the potential influence of cash transfers targeting the Eastern Band of Cherokee (measured by AI status) on the relation between the time exposed to the cash transfer and future birthing person/perinatal outcomes, uses a series of control populations to approximate a counterfactual expectation of birthing person and birth outcomes in the absence of the exposure.

We compare perinatal outcomes among AI birthing persons who were young children in 1996 to that of AI birthing persons who were older in 1996 (but still ≤18 years). Importantly, we also adjust for general cohort differences in access to social, educational, and economic resources in Jackson, Swain, and Graham counties. This cohort approach uses a comparison (control) group of non-AI persons who live in the same place as “treated” AI persons, and who are the same age as AI persons. The non-AI control group in this region, in our view, best matches the AI treatment group in terms of cohort experiences in this rural setting. Policy analysts and epidemiologists have employed this approach to examine the effect of large “shocks” on perinatal and child outcomes (e.g., Baker et al., 2019; Bleakley, 2007; Bruckner & Nobles, 2013).

In this study design, a key control group consists of non-AI birthing persons. We use this control group given the assumption that people living in the same counties have similar access to resources. Thus, we infer that the difference we can measure between AI and non-AI birthing persons lies in whether the cash transfer was received. This inference assumes that, absent the cash transfer, the difference in perinatal/maternal outcomes between AI and non-AI persons would have been consistent over time (Wing et al., 2018). Supplemental analyses, using outcomes that had no opportunity of the birthing person being exposed to the cash transfer as a child (i.e., mother was >18 years old before 1996), support this assumption (Table A1).

Estimation of the relation between duration of childhood exposure to family cash transfers and perinatal outcomes entails pooling data for AI and non-AI births in Jackson, Swain, and Graham counties, and regressing the outcomes on a dichotomous indicator capturing (1) AI race/ethnicity (as measured by birthing person's race/ethnicity from the Birth file), (2) a continuous indicator of childhood years remaining before age 18 at the start of the family cash transfer, and the two-way interaction term between AI race/ethnicity and childhood years remaining at the start of the family cash transfer (i.e., AI*duration). This interaction term captures the difference in the birthing person/birth outcome between AI birthing persons to residents who were young in 1996 and those who were older in 1996, *net of that same difference in non-AI birthing persons*.

The regression also includes controls for partner's (of birthing person) race/ethnicity and (depending on the outcome examined) parity, age and educational attainment of the birthing person. We applied ordinary-least-squares (OLS) regressions for continuous outcomes (i.e., age, educational attainment, pre-pregnancy BMI, and birthweight) and logistic regression for binary outcomes (i.e., preterm birth, tobacco use during pregnancy, SGA, and LGA). Given that many birthing persons have more than one birth over the test period, we estimated all standard errors using the “robust” option (using the *repeated subject* option in proc genmod in SAS, Cary, NC), which clusters observations by birthing person. We also conducted sensitivity tests that use clinically relevant categorical cutpoints and/or restrictions, recommended in the literature, for some outcomes (i.e., BMI (Weir & Jan 2022) and birthweight (Hughes et al., 2017)).

## Results

Our sample consists of 6805 children, of which 24% (i.e., 1647) were born to AI birthing persons (Table 1). AI birthing persons were, on average, two years younger at childbearing (vs. non-AI; 23.3 years vs. 25.2 years). Teen childbearing occurs more frequently for AI birthing persons (23.8% vs. 13.9% among non-AI in this sample). Additionally, AI birthing persons have relatively higher BMI prior to pregnancy (29.9 vs. mean BMI of 27.2 for non-AI). Furthermore, AI birthing persons were more likely to have less than high school education (30.5%) compared to non-AI birthing persons (21.8%). Infants born to AI birthing persons were relatively heavier with an average birthweight of 3387.8 g compared to non-AI birthing persons (mean = 3299.5 g).

**Fig. 1:**
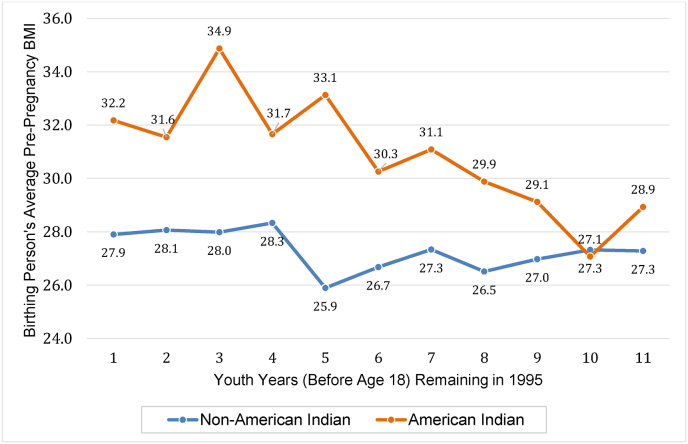
Birthing person's average pre-pregnancy BMI by youth years remaining in 1995 among AI and non-AI birthing persons in Jackson, Swain, and Graham County, 1997–2018.

Fig. 1 plots mean BMI—one outcome of interest which previous literature documents as responding to cash transfers—among AI birthing persons (Fernald, Gertler, & Hou, 2008, Rehkopf, Strully, & Dow, 2014). BMI appears to fall with increasing duration of exposure to the cash transfer during childhood. By contrast, BMI among non-AI birthing persons shows no relation with youth years remaining before age 18 (in 1995—see Fig. 1).

Results from the regression analyses (Table 2) show the relation to birthing person/birth outcomes of the interaction of birthing person's AI status and the number of years exposed during childhood to the cash transfer (full regression results available in Appendix Tables A2–A9). Each row represents a separate outcome and regression result. Among AI birthing people, age of childbearing (i.e., 0.15 years, 95% confidence interval [CI]: 0.05, 0.25) is positively associated with each additional year of exposure to family cash transfer during their childhood. When examining birthing person's age using categorical cutpoints, duration of the cash transfer is associated with a lower odds of teen childbearing (i.e., <20 years), but the odds ratio (OR) does not reach conventional levels of statistical detection (OR = 0.96; 95% CI: 0.92, 1.01). More years of childhood exposure to the cash transfer also vary positively with educational attainment among AI birthing persons, although this suggestive result does not reach conventional levels of statistical detection.

**Table 2:** Regression results^a^ predicting birthing person and birth outcomes for 6805 children in Jackson, Swain, and Graham County, 1997–2018, as a function of the interaction of AI race/ethnicity and duration of birthing person's exposure to family cash transfer as a child. Each row represents a separate regression.

Outcome Variable	Model 1		
	AI*duration Coefficient	95% CI	
Birthing Person's Age (Years)^b^	0.1489***	0.05	0.25
Birthing Person's Educational Attainment	0.0185*	−0.00	0.04
Birthing Person's BMI Pre-Pregnancy^c^^,^^d^	−0.4239**	−0.76	−0.09
Infant Birth Weight (in grams)^c^	−10.1652*	−22.1	1.81
	Odds Ratio for AI*duration	**95% CI**	
Tobacco Use during Pregnancy^c^ (y/n)	1.003	0.95	1.06
Preterm Birth^c^ (y/n)	1.005	0.94	1.08
Small-for-Gestational-Age^c^^,^^e^ (y/n)	0.89*	0.78	1.01
Large-for-Gestational-Age^c^^,5^ (y/n)	0.94*	0.88	1.00

(*p < 0.1, **p < 0.05, ***p < 0.01, ****p < 0.001).

a All models cluster observations by birthing person and include main effects of birthing person's race and duration of exposure to cash transfer.

b Includes partner's (of birthing person) race as covariate.

c Includes partner's (of birthing person) race, birthing person's age, birthing person's educational attainment, and parity as covariates. Restricted to singleton births only.

d Restricted to births between 2011 and 2018 (when BMI was recorded).

e Birthweight for gestational age below 10^th^ percentile; analysis restricted to 33 weeks gestational age or greater.

AI birthing persons with a longer duration of exposure to the cash transfer during childhood have lower pre-pregnancy BMI compared to expected levels (coef: −0.42, 95% CI: −0.76, −0.09). Consistent with the notion of lower per-pregnancy BMI, infants born to exposed AI birthing persons also show lower mean birthweight (coef. −10.17 g, 95% CI: −22.14, 1.81, p < 0.10). The odds of both SGA and LGA also are reduced among more exposed birthing persons/infants, which indicates a move toward the central tendency of fetal growth among more exposed birthing persons (p < 0.10). We, however, observe no relation between the duration of family cash transfer exposure and odds of preterm birth or tobacco use during pregnancy.

Additional sensitivity checks (Table 3) suggested that observed BMI and birthweight associations among the longer-exposed AI birthing persons reflected salutary changes. For the BMI analysis, we restricted the population to BMI >18.5 and used recommended clinically-relevant categorical cutpoints (rather than the continuous measure) (Doherty et al., 2006; Weir & Jan 2022). Consistent with results in Table 2, greater exposure to family cash transfer during childhood corresponds with reductions in categorical values of pre-pregnancy BMI (coef: −0.07, 95% CI: −0.12, −0.02).

**Table 3:** Regression results^a^ of sensitivity analyses predicting birthing person and birth outcomes for 6805 births in Jackson, Swain, and Graham County, 1997–2018, as a function of the interaction of AI race/ethnicity and duration of the birthing person's exposure to family cash transfer as a child. Each row represents a separate regression.

Outcome Variable	Model 1		
	AI*duration coefficient	95% CI	
Birthing Person's BMI Pre-Pregnancy^b^^,^^c^	−0.0688***	−0.1167	−0.0210
Infant Birth Weight (in grams)^d^	−10.1551*	−20.5781	0.2679

(*p < 0.1, **p < 0.05, ***p < 0.01, ****p < 0.001).

a All models cluster observations by birthing person and include main effects of birthing person's race and duration of exposure to cash transfer.

b Includes partner's (of birthing person) race, birthing person's age, birthing person's and educational attainment, and parity as covariates. Restricted to singleton births only.

c Restricted to births between 2011 and 2018 (when BMI was recorded) and to birthing person's BMI greater than 18.5.

d Restricted to births with birth weight greater than 2499 g.

We then reasoned that any potential reduction in birthweight among more exposed AI birthing persons might result in moving toward a lighter but healthy range of birthweight, rather than to low weight (<2,500 g) categories. We therefore repeated our birthweight analysis but restricted the test to births greater than 2500 g. The magnitude of birthweight result is very similar to that of the main analysis (coef: −10.16 g, 95% CI -20.58, 0.27), which lies just outside of conventional levels of statistical detection. The general shift to a healthier range of birthweight, however, does not correspond with a substantially reduced odds of macrosomia among more exposed AI birthing persons (OR = 0.98; 95% CI: 0.92, 1.04).

## Discussion

A previous cohort study of a southeastern AI tribe finds mental and physical health gains among adults exposed for a longer duration to a large family cash transfer when they lived at home as a child (Copeland et al., 2022). This prior work indicates that large income investments during childhood may promote health later in life. We extend this work to a larger population of AI birthing persons in this region and use the quasi-random timing of the large cash transfer to examine potential improvements in birthing person and perinatal outcomes among the next generation. AI birthing persons with greater duration of exposure to the cash transfer as a child show older age at childbearing, reduced pre-pregnancy BMI, lower odds of both SGA and LGA, and infants with slightly lower (but within a healthy range of) birthweight. Educational attainment also appears elevated among longer exposed birthing persons. We, by contrast, observe no relation between the duration of the cash transfer as children and birthing person's tobacco use or the risk of a preterm delivery. Taken together, in this rural AI population, cash transfers in one generation correspond with improved birthing person and infant health in the next generation.

To help the reader contextualize the size of the coefficients relative to other literature, an AI birthing person with ten years of exposure to the family cash transfer before age 18 years has, on average, a pre-pregnancy BMI of 4.2 points lower, and is 1.5 years older, than an AI birthing person with no exposure to the family cash transfer before age 18 years (per coefficients in Table 2). This BMI difference is similar to the average BMI gap between adult women in the US who graduated from college relative to women whose highest educational attainment was graduation from high school (Krishna et al., 2015).

Teenage childbearing among AI adults is almost two-fold more frequent than for non-AI adults in this rural population (Wingo et al., 2012). We observed a modest decrease in the risk of teenage childbearing among AI birthing persons with longer duration of exposure to the cash transfer. To the extent that the observed increase in age at childbearing among AI birthing persons reflects conscious fertility delay, such delayed timing may promote human capital in several ways. Prior research contends (with empirical support) that increased educational attainment can precede delays in childbearing (Cohen et al., 2011). In addition, persons who delay fertility into their mid-to-late 20s may increase their educational attainment and earnings and acquire a host of other experiences that might contribute to a healthier prenatal environment (Augustine et al., 2015; Duncan et al., 2018; Miller, 2011). Rigorous collection of these additional measures would further assist with a robust assessment of the potential merits of fertility delay on human capital.

BMI at the overweight (i.e., 25 to <30) and obese (i.e., 30 to <35) ranges substantially increases the risk of preeclampsia during pregnancy (He et al., 2020). Overweight and obese birthing person BMI also increase the risk of large for gestational-age weight at birth as well as offspring overweight/obesity and diabetes in childhood (Fang et al., 2019). The AI population we studied shows higher rates of overweight/obesity than other race/ethnicities. Our results indicate health benefits in this area in that family cash transfers early in childhood correspond with lower pre-pregnancy BMI and lighter infant birth weight among the infants of AI birthing persons. Longitudinal follow-up data of children in this region, such as that performed by the Great Smoky Mountains Study (GSMS), may further assist with identifying whether the observed reductions in pre-pregnancy BMI translate into lower diabetes and overweight/obesity in children, which appears especially elevated in AI populations nationwide (Bullock et al., 2017).

Strengths of our study involve the quasi-random timing of the onset of the cash transfer and use of a comparison population study design to minimize confounding. Results, for instance, cannot arise due to general improvements in AI health over the test period because we compared outcomes within AI pregnant women who differed according to their age when they were children in 1995. In addition, the use of a non-AI comparison group as well as sensitivity checks using data on persons unexposed to the cash transfer as a child (Table A1) makes it less likely that shared trends in the outcome (e.g., BMI) create spurious findings. Furthermore, as compared to smaller studies such as the GSMS cohort, ours has the advantage of using the entire population of the area and including birthing person and perinatal outcomes that do not rely on self-report. Taken together, the population-based approach and the cohort (GSMS) study on this cash transfer provide stronger evidence of AI health benefits via triangulation of evidence (Hammerton & Munafò, 2021).

Our study design also minimizes bias due to potential differences in social norms of AI and non-AI birthing persons. Non-AI communities may have different norms for obtaining higher education and delaying childbearing. Additionally, dietary patterns and physical activity levels have changed over time for AI communities (Lefler, 2009). When we compare AI birthing persons who are younger or older in 1995, such within-AI comparisons minimize the risk of confounding by such social norms.

For AI communities, there continues to be an educational disparity resulting in fewer high school graduates and consequently also fewer college graduates (Hunt & Harrington, 2010). AI communities living on tribal lands often live below the federal poverty line and receive inadequate health care. We, however, hesitate to speculate on the extent to which historical context and discrimination affected the contemporary EBCI case because we did not formally study these policies in this paper. In addition, we cannot know whether our study generalizes to other AI populations. We examined a single AI community with a singular history. The EBCI bought and own their land; they do not live on a reservation. In addition, the EBCI are comprised partly of descendants of Trail of Tears survivors and partly of descendants of Cherokee that hid out in the mountains and refused relocation. The EBCI likely experienced many social determinants that could have shaped their health (e.g., forced relocation, forced assimilation through boarding schools), but it is beyond the scope of this paper to explore the myriad reasons that AI communities more broadly have higher mean levels of poverty.

Owing to our reliance on the North Carolina vital statistics Birth File, limitations include the incompleteness of some variables (e.g., tobacco use during pregnancy) which may introduce measurement error in health behaviors. In addition, measurement errors and/or missingness of known paternity of the non-birthing partner (Anderson et al., 2016), as well as lack of information on the duration of cohabitation of the two parents, compelled us to consider AI race/ethnicity of the non-birthing parent as a covariate (rather than as an exposure measure to approximate the family value of AI cash transfer). Furthermore, the age of the AI individual at the start of the cash transfer (i.e., in 1996) is perfectly collinear with their “duration” of exposure to the cash transfer before adulthood. This collinearity implies that our work cannot determine whether age at initiation of cash transfer, or duration of cash transfer exposure, seems most relevant in predicting future perinatal outcomes of the birthing person. We also acknowledge that mean birthweight, while commonly used in the social sciences literature, does not have strong predictive value for later life morbidity and therefore should instead be interpreted as an indicator of the central tendency of fetal growth.

In addition, we could not rigorously examine potential life course pathways by which cash transfers during childhood may affect birthing person/perinatal outcomes later in life. Longitudinal studies of the GSMS cohort, however, provide some evidence of pathways. Associations of the cash transfer with behavioral health vary across stages of development, with initial associations before age 20 years on behavior symptoms, followed by associations with reduced substance misuse in early adulthood, and then reduced emotional symptoms in adulthood (ages 25 and 30 (Akee et al., 2010; Copeland et al., 2022; Costello et al., 2010)). These documented associations with emotional functioning provide a potential mechanism by which the longer duration of exposure to the cash transfer during childhood may reduce BMI for the birthing person (and reduce birth weight for the infant). We also note that a prior study in Canada finds that an unconditional cash transfer program precedes a reduction in birthing person BMI (Lebihan & Mao Takongmo, 2019), although the size of that cash transfer was less than 25% of the casino-based transfer in our AI population.

Chetty and colleagues (2014) find that, whereas income inequality has increased substantially in the US over the last 30 years, parents' income remains strongly correlated with their child's income when the child reaches age 26. This stability of intergenerational socioeconomic position implies that children born to low-income parents (i.e., in the lowest quintile) have a less than 10% probability of having an adult income at the top quintile once they reach age 26 (Chetty et al., 2017). By contrast, the introduction of the Harrah's casino on the EBCI lands dramatically lowered poverty in a short time period (Costello et al., 2016). We expect that this ecological change holds the potential to disrupt the general stability of intergenerational poverty in this population.

Whereas cash transfer programs may take quite diverse forms in terms of funding source, disbursement mechanism, target population, and eligibility (Aizer et al., 2022), unconditional cash transfer programs in the US tend to share some common elements. First, they tend to be means-tested in that only persons demonstrating a financial need (i.e., income below a specific threshold) would qualify. Second, the federal, state, or local governments typically serve as the funding source. Third, the unconditionality of the cash transfer means that persons can spend the funds in any way they choose. Fourth, the cash disbursement schedule often approximates that of receipt of labor market income (e.g., once every month). Each of these components may affect spending behavior and health differently (and sometimes in counterintuitive ways; see (Bruckner et al., 2013; Margerison et al., 2023)), which makes the structure of cash transfers an important area for ongoing research (Sun et al., 2021).

## Conclusion

A recent systematic review on cash transfers in the US finds mixed evidence about its relation with health, in that some work finds health improvements, while other reports show null associations or even adverse sequelae (Sun et al., 2021). We know of no prior work, however, that examines the relation between a child's exposure to cash transfers and subsequent birthing person/perinatal outcomes. We find that increased duration of cash transfer exposure during childhood is associated with an increase in age at childbearing and a decrease in pre-pregnancy body mass index. The odds of large-for-gestational age at delivery, as well as mean infant birthweight, is also reduced among AI births whose birthing person had relatively longer duration of exposure to the cash transfer. Although we hesitate to speculate on policy implications of this work to other populations, places, and times, our findings provide evidence that, for some perinatal outcomes, large cash transfers during childhood have the potential to improve health into the subsequent generation.

## Ethical statement

This study has been approved by Duke University Institutional Review Board (Protocol: Pro00090215).

## Declaration of interest statement

The authors have declared that no competing interests exist.

## CRediT authorship contribution statement

**Brenda Bustos:** Writing – review & editing, Writing – original draft, Visualization, Methodology, Formal analysis, Conceptualization. **Marcela Lopez:** Writing – review & editing, Writing – original draft, Visualization, Formal analysis. **Kenneth A. Dodge:** Writing – review & editing, Writing – original draft, Funding acquisition. **Jennifer E. Lansford:** Writing – review & editing, Writing – original draft, Funding acquisition. **William E. Copeland:** Writing – review & editing, Writing – original draft, Validation. **Candice L. Odgers:** Writing – review & editing, Writing – original draft, Validation. **Tim A. Bruckner:** Writing – review & editing, Writing – original draft, Methodology, Formal analysis, Conceptualization.

## Figures and Tables

**Table A1:** Falsification test using only unexposed birthing persons. Regression results^a^ predicting birthing person and birth outcomes for 6383 persons in Jackson, Swain, and Graham County as a function of the interaction of AI race/ethnicity and birthing person's adult age minus 18 years in 1996 (i.e., number of pre-exposure years). Only persons who were 18 years or older in 1996 are included in this falsification analysis. Each row represents a separate regression.

Outcome Variable	Model 1		
	AI*duration Coefficient	95% CI	
Birthing Person's Age (Years)^b^	−0.011	−0.14	0.12
Birthing Person's Educational Attainment	0.011	−0.01	0.03
Birthing Person's BMI Pre-Pregnancy^c^^d^	0.75	−0.77	2.17
Infant Birth Weight (in grams)^c^	−6.63	−20.82	7.55
	Odds Ratio for AI*duration	**95% CI**	
Tobacco Use during Pregnancy^c^ (y/n)	1.04	0.97	1.10
Preterm Birth^c^ (y/n)	1.005	0.92	1.10
Small-for-Gestational-Age^c^^4^ (y/n)	1.08	0.95	1.23
Large-for-Gestational-Age^c^^5^ (y/n)	1.004	0.95	1.06

(*p < 0.1, **p < 0.05, ***p < 0.01, ****p < 0.001).

a All models cluster observations by birthing person and include main effects of birthing person's race and duration of exposure to cash transfer.

b Includes partner's (of birthing person) race as covariate.

c Includes partner's (of birthing person) race, birthing person's age, birthing person's educational attainment, and parity as covariates. Restricted to singleton births only.

d Restricted to births between 2011 and 2018 (when BMI was recorded).

**Table A2:** Regression results of covariates predicting birthing person's age at childbirth (n = 6805) in Jackson, Swain, and Graham County, 1997–2018.

Variable	Birthing Person's Age		
	Estimate	95% CI	
Intercept	26.6288****	26.28	26.97
Birthing person's AI race/ethnicity (AI) (1/0)	−2.7156****	−3.47	−1.96
Partner's (of birthing person) AI race/ethnicity (1/0)	−0.3813*	−0.78	−0.02
Number of childhood years remaining after 1996 (*duration*)	−0.2582****	−0.31	−0.21
Interaction Term (AI x *duration*)	0.1489***	0.05	0.25

(*p < 0.1, **p < 0.05, ***p < 0.01, ****p < 0.001).

**Table A3:** Regression results of covariates predicting birthing person's education (n = 6805) in Jackson, Swain, and Graham County, 1997–2018.

Variable	Birthing Person's Education		
	Estimate	95% CI	
Intercept	1.2607****	1.20	1.32
Birthing person's AI race/ethnicity (AI) (1/0)	−0.3759****	−0.51	−0.24
Number of childhood years remaining after 1996 (*duration*)	0.0035	−0.01	0.01
Interaction Term (AI x *duration*)	0.0185*	−0.001	0.04

(*p < 0.1, **p < 0.05, ***p < 0.01, ****p < 0.001).

**Table A4:** Regression results of covariates predicting birthing person's BMI (n = 6805) children in Jackson, Swain, and Graham County, 1997–2018.

Variable	Birthing Person's BMI		
	Estimate	95% CI	
Intercept	22.4027****	18.64	26.16
Birthing person's age	0.1540***	0.05	0.26
Birthing person's education	−0.4027*	−0.85	0.04
Parity of birth	0.1029	−0.17	0.37
Birthing person's AI race/ethnicity (AI) (1/0)	5.9543****	3.31	8.60
Partner's (of birthing person) AI race/ethnicity (1/0)	0.7576	−0.31	1.82
Number of childhood years remaining after 1996 (*duration*)	0.0787	−0.08	0.24
Interaction Term (AI x *duration*)	−0.4239**	−0.76	−0.09

(*p < 0.1, **p < 0.05, ***p < 0.01, ****p < 0.001).

**Table A5:** Regression results of covariates predicting infant birthweight (n = 6805). in Jackson, Swain, and Graham County, 1997–2018.

Variable	Infant Birthweight		
	Estimate	95% CI	
Intercept	3267.624****	3177.25	3357.99
Birthing person's age	−0.5908	−4.50	3.32
Birthing person's education	59.8148****	36.61	83.02
Parity of birth	6.0844	−12.41	24.58
Birthing person's AI race/ethnicity (AI) (1/0)	149.3557***	68.51	230.20
Partner's (of birthing person) AI race/ethnicity (1/0)	43.1798*	−2.25	88.61
Number of childhood years remaining after 1996 (*duration*)	−3.5703	−9.44	2.30
Interaction Term (AI x *duration*)	−10.1652*	−22.14	1.8137

(*p < 0.1, **p < 0.05, ***p < 0.01, ****p < 0.001).

**Table A6:** Logistic Regression results of covariates predicting birthing person's tobacco use during pregnancy (n = 6805) in Jackson, Swain, and Graham County, 1997–2018.

Variable	Birthing Person's Tobacco Use		
	Estimate	95% CI	
Intercept	0.38****	0.26	0.56
Birthing person's age	0.99	0.98	1.01
Birthing person's education	0.59****	0.54	0.65
Parity of birth	1.33****	1.24	1.41
Birthing person's AI race/ethnicity (AI) (1/0)	0.92	0.63	1.33
Partner's (of birthing person) AI race/ethnicity (1/0)	0.92	0.75	1.12
Number of childhood years remaining after 1996 (*duration*)	1.007	0.98	1.03
Interaction Term (AI x *duration*)	1.003	0.95	1.06

(*p < 0.1, **p < 0.05, ***p < 0.01, ****p < 0.001).

**Table A7:** Logistic Regression results of covariates predicting preterm birth (n = 6805) in Jackson, Swain, and Graham County, 1997–2018.

Variable	Preterm Birth		
	Estimate	95% CI	
Intercept	0.07****	0.04	0.13
Birthing person's age	1.01	0.99	1.04
Birthing person's education	0.94	0.82	1.08
Parity of birth	1.05	0.93	1.19
Birthing person's AI race/ethnicity (AI) (1/0)	1.006	0.61	1.65
Partner's (of birthing person) AI race/ethnicity (1/0)	0.85	0.64	1.13
Number of childhood years remaining after 1996 (*duration*)	1.002	0.97	1.04
Interaction Term (AI x *duration*)	1.005	0.94	1.08

(*p < 0.1, **p < 0.05, ***p < 0.01, ****p < 0.001).

**Table A8:** Logistic Regression results of covariates predicting small for gestational age (n = 6805) in Jackson, Swain, and Graham County, 1997–2018.

Variable	Small for Gestational Age		
	OR	95% CI	
Intercept	0.07****	0.02	0.25
Birthing person's age	1.00	0.94	1.06
Birthing person's education	0.75**	0.59	0.96
Parity of birth	0.76**	0.59	0.97
Birthing person's AI race/ethnicity (AI) (1/0)	1.98**	0.94	4.18
Partner's (of birthing person) AI race/ethnicity (1/0)	1.14	0.72	1.79
Number of childhood years remaining after 1996 (*duration*)	1.03	0.96	1.10
Interaction Term (AI x *duration*)	0.89*	0.78	1.01

(*p < 0.1, **p < 0.05, ***p < 0.01, ****p < 0.001).

**Table A9:** Logistic Regression results of covariates predicting large for gestational age (n = 6805) in Jackson, Swain, and Graham County, 1997–2018.

Variable	Large for Gestational Age		
	OR	95% CI	
Intercept	0.09****	0.05	0.19
Birthing person's age	1.03	0.99	1.06
Birthing person's education	1.25****	1.10	1.42
Parity of birth	1.03	0.92	1.16
Birthing person's AI race/ethnicity (AI) (1/0)	2.10****	1.42	3.09
Partner's (of birthing person) AI race/ethnicity (1/0)	1.09	0.86	1.39
Number of childhood years remaining after 1996 (*duration*)	1.01	0.98	1.05
Interaction Term (AI x *duration*)	0.94*	0.88	1.00

(*p < 0.1, **p < 0.05, ***p < 0.01, ****p < 0.001).

## Data Availability

The authors do not have permission to share data.
